# The Frequency of Focal Thyroid Incidental Findings and Risk of Malignancy Detected by 18F-Fluorodeoxyglucose Positron Emission Tomography in an Iodine Deficient Population

**DOI:** 10.3390/diagnostics8030046

**Published:** 2018-07-17

**Authors:** Nina Gedberg, Jesper Karmisholt, Michael Gade, Rune V. Fisker, Victor Iyer, Lars J. Petersen

**Affiliations:** 1 Department of Otolaryngology, Aarhus University Hospital, DK-8000 Aarhus, Denmark; ninagedbjerg@gmail.com; 2 Department of Endocrinology, Aalborg University Hospital, DK-9000 Aalborg, Denmark; jsk@rn.dk; 3 Department of Clinical Medicine, Aalborg University, DK-9000 Aalborg, Denmark; 4 Department of Nuclear Medicine, Clinical Cancer Research Centre, Aalborg University Hospital, DK-9000 Aalborg, Denmark; michael.g@rn.dk (M.G.); rvf@rn.dk (R.V.F.); petctiyer@gmail.com (V.I.); 5 Department of Radiology, Aalborg University Hospital, DK-9000 Aalborg, Denmark

**Keywords:** Denmark, incidental findings, iodine deficiency, neoplasms, positron emission tomography, thyroid

## Abstract

Incidental focal uptake of 18F-fluorodeoxyglucose (FDG) in the thyroid on positron emission tomography (PET/CT) is rare but often associated with malignancy. The epidemiology of thyroid incidentalomas has only to some extent been described in countries with iodine deficiency. Here we report data from Denmark, a country with known iodine deficiency and wide access to PET/CT. All FDG PET/CT comprising the head and neck region, during 2014, were retrospectively reviewed, and patients with focal FDG uptake in the thyroid gland were identified. A total of 2451 patients had an FDG PET/CT of which 59 (2.4%) patients presented with FDG-avid focal lesions in the thyroid gland. Among the 59 patients with FDG-avid lesions, 33 patients (56%) received work up with ultrasound, thyroid technetium scintigraphy, fine needle aspiration, and/or histology of which 20 patients had a conclusive pathology report. Ten patients with FDG-avid lesions were identified with thyroid malignancy. The risk of thyroid malignancy was 16.9% among patient with incidental FDG-avid thyroid lesions. Our findings indicated a similar frequency of FDG thyroid incidentalomas and malignancy rates in an iodine deficient population compared to summary data from prior studies, studies mostly performed in geographical areas of normal or excess iodine supplementation.

## 1. Introduction

Incidental imaging findings in a tissue or organ in patients without signs or symptoms of disease in that tissue (incidentalomas) are frequently occurring [1]. Incidental findings with 18F-fluorodeoxyglucose positron emission tomography/computer tomography (FDG PET/CT) has been reported in several tissues [2], including colon [3], prostate [4], breast [5], and the adrenal glands [6].

Thyroid incidentalomas are common in imaging, but the risk of an underlying malignancy varies greatly depending on the imaging modality used [7] and the proportion of thyroid nodules in the background population [8]. A notable number of studies and systematic reviews have investigated the frequency and malignancy rate of FDG-avid thyroid incidentalomas in PET/CT [9,10,11,12]. A total of 32 original retrospective reports showed a median frequency of 2.3% of FDG-avid lesions in the populations studied, with thyroid cancer in nearly one in every three patients with focal FDG-avid thyroid lesions with follow up [12].

Most of the current retrospective studies on FDG incidentalomas have been performed in countries with normal or excess iodine supply, e.g., USA and South Korea [12,13,14]. Iodine deficiency is present in several parts of the World, including many areas of Europe (e.g., Scandinavia), Middle East, and Africa [14,15,16]. Low iodine supply may cause a high proportion of thyroid nodules in the population [8]. Only a few papers on FDG-avid thyroid incidentalomas have been published from iodine-deficient areas of Europe, namely two papers from Italy [17,18], one from UK [19] and one from Sweden [20]. These papers have consistently reported frequencies of 1.0–1.8% for FDG-avid focal lesions, well below the median frequency of 2.3% among the 32 currently published papers [12]. In contrast, the reported malignancy rates among patients with focal thyroid FDG uptake and subsequent follow-up in these four papers ranged from 23% in the UK, 34–43% in Italy, to 59% in Sweden compared to a median proportion of 32% among 32 papers [12].

Several issues warrant additional studies on the epidemiology and malignancy rates of FDG-avid incidentalomas. First, more studies are required from areas with low iodine supplementation due to conflicting data of the scarce published reports. In Denmark, up to 39% of women in the age 60–65 years have one or more thyroid nodules over 10 mm on ultrasound [21], but thyroid cancer is relatively infrequent [22]. Second, the access to PET/CT, and thereby the patient population referred for FDG PET/CT, varies greatly, even among European countries [23]. The number of PET/CT scanners and PET/CT examinations per inhabitants in Denmark are among the highest in the World [24]. The purpose of this work was to investigate the frequency and malignancy rates of focal, FDG-avid thyroid lesions in an iodine-deficient population with wide access to FDG PET/CT.

## 2. Materials and Methods

### 2.1. Patients

We retrospectively reviewed all FDG PET/CT that included the head and neck region and were performed at our institution from 1 January to 31 December 2014. Our department performed PET/CT for all patients in the Region North, Denmark; an area covering approximately 600,000 inhabitants. Patients were included in this study if the PET report identified focal FDG-uptake confined to the thyroid gland, excluding patients with diffuse thyroid FDG uptake. Patients with known malignant thyroid disease at the time of the PET/CT, as well as patients with a recent fine needle aspiration of the thyroid were excluded, so were patients with focal FDG uptake in the thyroid shown in any prior PET/CT. From the electronic medical record system, we registered data from thyroid ultrasound, thyroid scintigraphy, clinical follow-up, and the description of the pathological sample from the thyroid (fine needle aspiration of the lesion, pathological examinations of surgical specimens). The data registration was performed for at least 24 months after the PET/CT and was terminated in October 2017. Any patients with thyroid malignancy proven by pathology within 24 months from the PET/CT were registered as thyroid malignancy in our study. The Bethesda reporting system for cytopathology was used [25].

### 2.2. Imaging Details

The PET/CT was performed on a Discovery VCT scanner (GE Healthcare, Waukesha, WI, USA) in accordance with institutional procedures. The mean activity of FDG activity was 370 MBq. Blood glucose levels were less than 11 mmol/L in all patients. The PET/CT was acquired approximately 60 min after tracer injection. The CT scan was performed as low-dose CT or diagnostic CT with contrast-enhancement.

Classification of pathological FDG uptake in the thyroid was based on clinical impression (relative uptake in the thyroid versus background and/or asymmetry) and supported by semi-quantitative analysis of the uptake by calculation of standardized uptake value (SUV). Per institutional practice, all FDG PET/CT scans were read independently by two trained hybrid imaging specialists, in most cases a nuclear medicine physician and a radiologist, and a final diagnosis of pathological FDG uptake was made in consensus. For this study, the CT scans were used exclusively to verify the thyroidal confinement of the FDG-avid lesions.

### 2.3. Approvals

This quality assurance study was approved by the Danish Data Protection Agency at Region Nordjylland and covered by the general approval obtained by the region (2008-58-0028). The approval carried a waiver for informed consent to medical files. Retrospective studies do not require approval by Ethical Committees according to national legislation.

### 2.4. Statistics

All statistical tests were performed using SPSS ver. 24.0 (SPSS IBM, Armonk, NY, USA). Summary data are expressed as number and percentage, mean ± standard deviation (SD) or median (range), depending on the distribution of data.

## 3. Results

A total of 2451 patients had a PET/CT covering the head and neck region of which 59 (2.4%) patients presented with an incidental, focal FDG-avid lesion in the thyroid (Table 1). The mean age of patients with FDG-avid lesions was 69 years, nearly 70% of patients were women, many patients had a known non-thyroidal cancer at the time of referral, and patients were referred from a variety of medical specialities. The reason for PET/CT was cancer in most patients (95%), equally distributed among patients with known cancer and patients suspected of cancer or recurrent cancer.

Approximately 50% of the patients (*n* = 33) had imaging and/or pathology follow-up, mostly with ultrasound. Twenty-one patients had cytology, pathology or both of which 20 (34%) patients had a definitive cytological or histological diagnosis. One patient was diagnosed with a benign colloid nodule at autopsy. During a follow up period of two years, ten patients were diagnosed with thyroid cancer (nine cases of papillary carcinoma and one case of medullary carcinoma). Ten patients had benign pathology (eight cases of colloid nodules and two cases of thyroiditis). There were no cases of late thyroid malignancy (>2 years after the PET/CT) in any patients.

The proportion of pathology-proven thyroid malignancy was 16.9% among patients with FDG-avid thyroid lesions, 30.3% among patients with FDG-avid lesions who received follow-up, and 50.0% among patients with pathology as reference test (Table 2). Summary data from a recent systematic review is tabulated in Table 2 for comparison [12].

**Figure 1:**
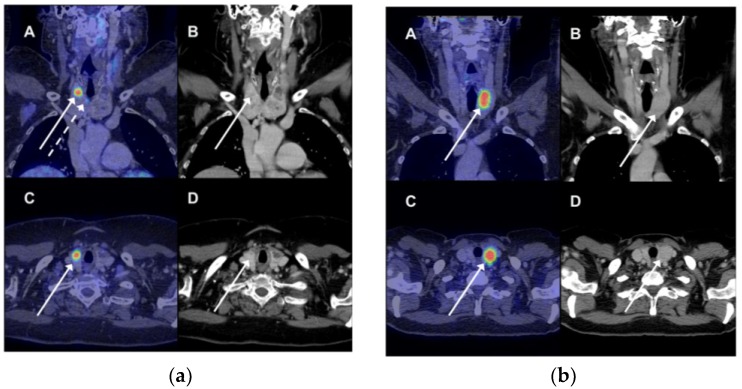
(**a**) A 69-year woman had a 18F-fluorodeoxyglucose positron emission tomography/computer tomography (FDG PET/CT) scan because of suspected pancreatic cancer (not verified). There was incidental FDG uptake in three lesions in the right thyroid. The patient underwent total thyroidectomy; pathology showed papillary adenocarcinoma in all three PET-avid lesions. (A) Fused coronal PET/CT images showed intense FDG uptake (standardized uptake value, SUV 9.8) in the upper right part of the right thyroid (full white arrow). Another site of pathological FDG uptake was seen on this coronal slice as well (dotted white arrow). (B) The CT image showing a bilateral nodular goiter with an arrow indicating the site of most intense FDG uptake on the fused images. (C + D) Corresponding transverse images of the fused PET/CT images and CT scan with the most intense FDG-avid lesion; (**b**) An FDG PET/CT was performed in a 69-year woman due to suspicion of ovarian cancer (confirmed). Incidental FDG uptake was seen in the left lobe. A fine needle aspirate showed benign findings (colloid nodule). Any false-negative findings were ruled out during clinical and imaging follow. (A) Fused coronal PET/CT images showed intense FDG uptake (SUV 11.0) in the upper right part of the right thyroid (full white arrow). (B) The CT image showing a bilateral nodular goiter with an arrow indicating the most intense site of pathological FDG uptake on the fused images. (C + D). Corresponding transverse images of the fused PET/CT images and CT scan of the most intense FDG-avid lesion.

Among those 26 patients without follow-up, 21 (84%) patients had a diagnosis of cancer at the time of the PET/CT or was diagnosed with cancer during the follow-up period. Thirteen patients died from non-thyroid cancer within one year of the PET/CT. None of the 26 patients were diagnosed with thyroid malignancy at the end of the observation period. There was no follow-up for thyroid malignancy among the 2000 or more patients without FDG-avid thyroid lesions. Illustrative examples of focal FDG uptake are shown in Figure 1.

Thirty-five patients had a non-thyroid cancer at the time of the PET/CT (Table 1, 29 patients with known cancer referred for PET and 6 patients with suspected recurrence), 13 patients were diagnosed with malignancy after the PET/CT, including 10 patients with thyroid malignancy, whereas 11 (18.6%) patients were not diagnosed with any cancer within the observation period after PET/CT.

## 4. Discussion

Incidental focal increased uptake of FDG in the thyroid on PET/CT has been described in a number of studies. However, there is a very limited number of publications from countries with documented iodine-deficiency, and these reports have shown conflicting results. To what extent wide access to PET/CT had any influence on the epidemiology of FDG PET/CT incidentalomas are not previously reported. We assessed the epidemiology of FDG-avid thyroid lesions in Denmark, a population deficient of iodine and an unparalleled access to FDG PET/CT for oncology and non-oncology indications. We concluded that the proportion of FDG-avid thyroid nodules and the malignancy rates among the patients in this study were quite similar to data from studies performed in the other regions of the World.

The key findings in this report were demonstration of FDG-avid lesions in 2.4% of the total population and the malignancy rates, which were 17% in patients with FDG-avid lesions, 30% among patients with focal FDG-avid lesions and follow-up, and 50% in patients who had a conclusive cytology/pathology examination. These figures are very similar to data presented in recent systematic reviews on thyroid incidental findings by FDG PET/CT [10,11,12]. Prior reports seldom present data among patients with a final pathology examination. These data are available in this report. Absence of a final cytological and/or pathological reference, as seen in approximately one-third of the patients, was likely caused by ultrasound and/or thyroid scintigraphy findings without suspected nodules.

It is remarkable that only approximately fifty percent of the patients with FDG-avid lesions received follow-up by ultrasound, thyroid scintigraphy and pathology examination by fine needle aspiration and/or surgical specimen examination. These findings likely reflect that the majority of the patients had, or subsequently were diagnosed with, non-thyroid malignancy at an advanced stage and died within the observation period. Our data confirmed a 50% cancer mortality rate within one year from the PET/CT in patients without work-up, and a cancer diagnosis in nearly 85% in these patients. The follow-up data are in line with data from previously published reports as shown in Table 2 [12].

It remains unclear if data on the epidemiology and malignancy rates of FDG-avid thyroid lesions are different in geographical areas with iodine-deficiency compared to no deficiency. Most published studies come from areas with normal or excess iodine supply. We found that 2.4% of the patients presented with FDG-avid focal lesions, which is similar to data from all regions published in systematic reviews, as shown in Table 2. However, the frequency of FDA-avid lesions in this report was higher than previously reported (1.0–1.8%) from other iodine-deficient European countries like Italy [17,18], UK [19], and Sweden [20]. The malignancy rates among patients with FDG-avid lesions undergoing follow-up was 30% in this study compared to 34–42% in Italy, 23% in the UK, and 59% in Sweden. The reasons for this large variation remain speculative, but the composition of the patient population may be important. Denmark has a very high number of PET/CT scanners, adjusted for population size, and wide access to PET/CT via free public hospital care without insurance or reimbursement issues [24]. Less than 60% of the patients in this study had pathology-verified cancer at the time of the PET/CT, and approximately one in every five patients were not diagnosed with cancer within a follow-up period of 3 years from the PET. The other studies from iodine-deficient countries included FDG-avid patients with cancer only [18,20] or included patients with known, as well as suspected cancer and/or infection without providing specific data by indication for a patient with focal FDG uptake [17,19]. Finally, the duration of the follow-up period was infrequently described in prior papers. Even though most thyroid cancers were detected in close relation to the initial work-up, we identified one patient with thyroid cancer, which was missed during the initial work-up but diagnosed within two years from the PET/CT.

The prevalence of thyroid nodules may notable influence the prevalence of thyroid incidentalomas, including FDG-avid lesions. Variation on thyroid nodule prevalences may interfere with epidemiological data on the frequency and malignancy rates of FDG-avid lesions. Nation-wide iodine fortification programs have generally been in place for many years in many iodine-deficient areas, including Denmark where it was initiated in 1997 [8,26]. However, recent follow-up data showed an inconsistent effect in such programs on iodine excretion, as well as thyroid volume changes [27,28]. Therefore, we as imaging specialists may expect to observe thyroid incidentalomas regularly in the years to come.

This study reported the frequency of thyroid incidental findings on FDG PET/CT performed as part of normal clinical practice. Two specialists always read the FDG PET/CT, and the conclusion was a consensus decision. We always had a radiologist trained in FDG PET to assist in the reading; in this context, the anatomical reading was of minimal importance, since the CT was mainly used to assure anatomical localization of incidental findings to the thyroid. The study did not examine observer variation in the identification of FDG-avid thyroid lesions; the study presented clinical findings as these were accessible to the referring physician.

Attempts have been made to identify the risk of thyroid malignancy among patients with FDG-avid lesions, e.g., lesion size and SUV. Such investigations have shown notable variation in SUV values among groups with malignant and benign thyroid lesions [18,19,20]. So far, the predictive values of individual variables remain uncertain. We did no calculations of SUV and/or sizes of the thyroid lesions. In addition, we did not measure the size of the thyroid incidentalomas, so any recommendations based on lesion size would be speculative.

## 5. Conclusions

In conclusion, we found a low frequency of incidental thyroid lesions with focal increased uptake of FDG on PET/CT. However, the malignancy rate was high in patients with follow-up (30%), reaching 50% in patients with pathology of the thyroid. Several guidelines recommend work-up of FDG-avid incidental findings by ultrasound [29,30]. The high rate of thyroid malignancy in patients with focal FDG-avid lesions suggest that a work-up of such lesions should include at least a fine needle aspiration cytology.

## Figures and Tables

**Table 1:** Patient demographics, follow up, and final pathological diagnosis among the 59 patients with FDG-avid focal thyroid uptake.

Variable	Data
Number of patients	59
Age (mean ± SD)	68 ± 9 years
Sex distribution	40 females (68%), 19 males (32%)
Known, non-thyroidal cancer at referral	35 (59%)
Cause of FDG PET/CT	
Known cancer (pathology-verified)	29 (49%)
Staging	16
Therapy monitoring	6
Cancer of unknown primary	7
Suspected cancer	27 (46%)
Suspected recurrence	6
Suspected cancer	21
Non-cancer (infection)	3 (5%)
Referring medical specialties	
Pulmonary medicine	20 (34%)
Surgical gastroenterology	13 (22%
Oncology/Hematology	9 (15%)
Gynecology	5 (8%)
Otolaryngology	4 (7%)
Other	8 (14%)
Imaging or pathology follow-up	33 (56%)
Thyroid ultrasound	31
Fine needle aspiration	17
Thyroid scintigraphy	15
Surgery	13
Autopsy	1

Abbreviations: FDG PET/CT: 18F-fluorodeoxyglucose positron emission tomography/computer tomography; SD: Standard deviation.

**Table 2:** Main findings of this study compared to summary data from 32 retrospective papers presented in a recent systematic review by Asmar et al. [12].

Populations	This Study	Asmar et al., 2017 (Median and Range) *
Study population, *n*	2451	4330 (32–49,519)
Patients with incidental, focal FDG-avid thyroid lesions, *n*	59	80 (19–1.151)
Proportion of patients with FDG-avid thyroid lesions	2.4%	2.3% (0.1–10.1)
Patient with follow up, *n*	33	38 (11–211)
Proportion of patients with follow up	56%	48% (11–100)
Patients with a conclusive pathology report, *n*	20	
Patients with proven thyroid malignancy, *n*	10	12 (3–72)
Thyroid malignancy data		
Malignancy among patients with FDG-avid lesions	16.9%	13.7% (2.5–55.0)
Malignancy among patient with FDG-avid lesions and follow-up	30.3%	31.5% (12.4–63.6)
Malignancy among patients with FDG-avid lesions and conclusive pathology	50.0%	Not reported

Abbreviation: FDG: 18F-fluorodeoxyglucose. * Data are captured from individual reports listed in Asmar et al., 2017 and summarized.
